# Major HPB Procedures Must be Undertaken in High Volume Quaternary Centres?

**DOI:** 10.1155/2000/52097

**Published:** 2000-01

**Authors:** A. N. Kingsnorth

**Affiliations:** Postgraduate Medical School Department of Surgery, Derriford Hospital Level 7, Plymouth UK


*HPB Surgery*, 2000, Vol. 11, pp. 359–361
Reprints available directly from the publisher
Photocopying permitted by license only

© 2000 OPA (Overseas Publishers Association) N.V.
Published by license under
the Harwood Academic Publishers imprint,
part of The Gordon and Breach Publishing Group.
Printed in Malaysia.

**HPB INTERNATIONAL**

**EDITORIAL & ABSTRACTING SERVICE**

JOHN TERBLANCHE EDITOR
Department of Surgery,
Medical School · Observatory 7925
Cape Town · South Africa
Telephone: 27-21-406-6204
Telefax 27-21-448-6461
E-mail: jterblan@uctgsh1.uct.ac.za

### Major HPB Procedures Must be Undertaken in High Volume Quaternary Centres?

### ABSTRACT

*Birkmeyer, J. D., Samuel, R. G., Finlayson, M. P. H., Tosteson, A. N. A., Sharp, S. M., Warshaw, A. L. and Fisher, E. S.*

*Background:* Reports of better results at national referral centers than at low-volume community hospitals have prompted calls for regionalizing pancreaticoduodenectomy (the Whipple procedure). We examined the relationship between hospital volume and mortality with this procedure across all US hospitals.

*Methods:* Using information from the Medicare claims database, we performed a national cohort study of 7229 Medicare patients more than 65 years old undergoing pancreaticoduodenectomy between 1992 and 1995. We divided the study population into approximate quartiles according to the hospital's average annual volume of pancreaticoduodenectomies in Medicare patients: very low (<1/*y*), low (1–2/*y*), medium (2–5/*y*), and high (5+/*y*). Using multivariate logistic regression to account for potentially confounding patient characteristics, we examined the association between institutional volume and in-hospital mortality, our primary outcome measure.

*Results:* More than 50% of Medicare patients undergoing pancreaticoduodenectomy received care at hospitals performing fewer than 2 such procedures per year. In-hospital mortality rates at these low- and very-low-volume hospitals were 3- to 4-fold higher than at high-volume hospitals (12% and 16%, respectively, *vs.* 4%, $P<.001$). Within the high-volume quartile, the 10 hospitals with the nation's highest volumes had lower mortality rates than the remaining high-volume centers (2.1% *vs.* 6.2%, $P<.01$). The strong association between institutional volume and mortality could not be attributed to patient case-mix differences or referral bias.

*Conclusions:* Although volume-outcome relationships have been reported for many complex surgical procedures, hospital experience is particularly important with pancreaticoduodenectomy. Patients considering this procedure should be given the option of care at a high-volume referral center. (*Surgery*, 1999, 125, 250–6.)

From the Center for the Evaluative Clinical Sciences and the Departments of Medicine and Surgery, Dartmouth Medical School, Hanover, NH, the Veterans Administration Outcomes Group, Department of Veterans Affairs Medical Center, White River Junction, Vt, and the Department of Surgery, Massachusetts General Hospital, Boston, Mass

*Keywords:* Pancreaticoduodenectomy, hospital mortality

### PAPER DISCUSSION

In the last decade a number of studies have concluded that hospital volume favourably influences the results of pancreaticoduodenectomy and of equal importance, that low volume results in higher mortality. The problem however is not that simple. Some of the facts within Birkmeyer's study need to be re-stated. First, following pancreaticoduodenectomy in patients over the age of 65 in the United States the overall postoperative mortality is 11.1%. Second, the resectability rate for pancreatic cancer in the United States (calculated on the basis of 1800 resections per year in patients over 65 equivalent to a total resection rate of 3600 from a total annual incidence of 29,000 pancreatic cancers) is 12%. Third, from 4 publications the authors have quoted very favourable 5-year survival rates ranging from 10% – 25%. They have not discussed the contentious paper by Gudjonsson that dealt with survival rates after resection for carcinoma of the pancreas. Gudjonsson standardised and compared results reported in 340 publications and reported that the best overall 5-year survival rate in a surgical series was only 3.6% [1].

Contrary to the authors suggestion that only State-level studies have been performed previously in analysis of pancreaticoduodenectomy results, a number of national and international surveys can be found in the literature. Glazer and colleagues analysed 28 series and a total of 2,172 patients undergoing pancreatic resection and found 150 deaths (6.9%) with a median mortality of 7.5% and an interquartile range of 4.5% – 12% [2, 3]. Janes and colleagues on behalf of the commission on cancer of the American College of Surgeons, conducted a large national survey to assess methods of diagnosis, staging, treatment and outcome of patients with adenocarcinoma of the pancreas: in two study periods involving 987 institutions and nearly 17,000 cases, they reported a lower operative mortality in units performing more than 20 cases per year [4]. Neoptolemos and colleagues on behalf of the UK Pancreatic Cancer Group analysed 1,026 resections of pancreatic and periampullary tumours in institutions performing 5 – 7 resections per year and revealed an operative mortality of 5% [5]. Finally, a recent publication by Simunovic and colleagues, who investigated postoperative mortality after pancreatic resection in Canadian publicly funded hospitals, reported a 3.4% mortality in high volume hospitals *i.e.*, those performing more than 42 resections in the 6-year period of the study [6], compared with a 14.4% mortality in low volume hospitals.

Such international and national surveys appear to reinforce the high volume: low mortality message. However, another important publication has been omitted from the authors argument, which is the paper by Wade and colleagues relating to the treatment of periampullary cancer in the US Department of Defence hospitals [7]. In this study the 30-day operative mortality rate was 8.5% and was equivalent in both teaching hospitals and smaller community type institutions. The highest volume hospital performed 3 – 4 resections per year and had similar mortality rates to smaller institutions performing fewer resections. The authors of this paper argued that the even distribution of mortality was due to the lack of financial and logistic barriers in the smaller hospitals, which enabled them to maintain facilities and staffing to a comparable standard in both the larger teaching hospitals and the smaller community hospitals. This argument has been addressed briefly by Berkmeyer and colleagues but discounted because they believe that quality improvements in hospitals performing low numbers of pancreaticoduodenectomies per year would be inefficient and impossible to evaluate at the local level. Such an attitude would impoverish smaller hospitals at the expense of larger institutions and further impair the ability of small hospitals to perform quality surgery. Also, at what point are the "underperforming" hospitals eliminated from practising complex surgery? And how many times will the cycle be repeated until all complex surgery is performed in super-institutions? Moreover it has been demonstrated that once a quality service has been established by investment in capital and personnel, the cost to the health care system of performing each pancreaticoduodenectomy diminishes because morbidity and mortality is lowered [8, 9]. Or, put more simply in order to save money (and lives) capital investment must be made, not only in equipment but also in personnel and training.

Patterns of work may also be important. Considering hospital volume, the high volume hospitals will have a larger number of surgeons performing pancreaticoduodenectomies and therefore the number of cases per surgeon may be similar to the cases per surgeon in the smaller hospitals. In the larger institutions, 2 specialist surgeons probably work together performing a 6–8 hour pancreaticoduodenectomy operation and therefore, may be able to eliminate errors due to fatigue and inattention. Thus an investment in teams of surgeons that are available to work together in the operating room and in the pre- and postoperative care of the patient in conjuction with a team of gastroenterologists, radiologists and intensivists is probably the correct path to follow to reduce mortality. In practical terms, there may be a compromise between the wishes of the patient to stay near to their home for their major surgery and the health care planners who will see the need to have low mortality in a quality environment.

An imaginative means of upgrading quality in smaller hospitals by the use of Telemedicine is suggested by Birkmeyer and colleagues. This solution readily lends itself to radiology but is less practical for the surgeon on the spot or the intensivist faced with a rapidly deteriorating patient in the early postoperative period. More power could be put in the hands of the patients by publishing pancreaticoduodenectomy mortality rates by institution which would enable the public to "vote with their feet" and encourage hospitals with high mortality to make the necessary investments to improve their results.

Analysis of surgical outcome is complex and is rarely dependent on one variable; we need to investigate the results of pancreaticoduodenectomy by more than hospital volume alone [10].

A. N. Kingsnorth BSc MS FRCS FACS
Postgraduate Medical School
Department of Surgery, Derriford Hospital
Level 7, Plymouth
U.K.

### *References*

[1] Gudjonsson B. (1995). Carcinoma of the pancreas: critical analysis of costs, results of resections and the need for standardized report. *J. Am. Coll. Surg.*, **181**, 483–503.

[2] Glazer, G., Coulter, C., Crofton, M. E. *et al.* (1995). Controversial issues in the management of pancreatic cancer: Part one. *Ann. R. Coll. Surg. Engl.*, **77**, 111–122.

[3] Glazer, G., Coulter, C., Crofton, M. E. *et al.* (1995). Controversial issues in the management of pancreatic cancer; Part two. *Ann. R. Coll. Surg. Engl.*, **77**, 174–180.

[4] Janes, R. H., Niederhuber, J. E., Chmid, J. S. *et al.* (1996). National patterns of care for pancreatic cancer: results of a survey by the commission on cancer. *Ann. Surg.*, **223**, 261–272.

[5] Neoptolemos, J. P., Russell, R. C. G., Brownhall, S. and Theis, B. (1997). Low mortality following resection for pancreatic and periampullary tumours in 1026 patients: UK survey of specialist pancreatic units. *Br. J. Surg.*, **84**, 1370–1376.

[6] Simunovic, M., To, T., Theriautt, M. and Langer, B. (1999). Relations between hospital surgical volume and outcome for pancreatic resection for neoplasm in a publicly funded health care system. *CMAJ*, **160**, 643–648.

[7] Wade, T. P., Coplin, M. A., Virgo, K. S. and Johnson, F. E. (1994). Periampullary cancer treatment in US Department of Veterans Affairs Hospitals: 1987–1991. *Surgery*, **116**, 819–826.

[8] Gordon, T. A., Burleyson, G. P., Tielsch, J. M. and Cameron, J. L. (1995). The effects of regionalization on cost and outcome for one general high-risk surgical procedure. *Ann. Surg.*, **221**, 43–49.

[9] Holbrook, R. F., Hargrave, K. and Traverso, W. (1996). A prospective cost analysis of pancreatoduodenectomy. *Am. J. Surg.*, **171**, 508–511.

[10] Kingsnorth, A. N. (1997). Surgery for periampullary and pancreatic carcinoma: a Liverpool experience. *Ann. R. Coll. Surg. Engl.*, **79**, 259–263.

